# Soil Microbial Community Characteristics and Their Effect on Tea Quality under Different Fertilization Treatments in Two Tea Plantations

**DOI:** 10.3390/genes15050610

**Published:** 2024-05-11

**Authors:** Yu Lei, Ding Ding, Jihua Duan, Yi Luo, Feiyi Huang, Yankai Kang, Yingyu Chen, Saijun Li

**Affiliations:** 1 Tea Research Institute, Hunan Academy of Agricultural Science, Changsha 410125, China; leiyu0727@163.com (Y.L.); dingding881103@163.com (D.D.); duanjihua09@163.com (J.D.); luoyi197912@163.com (Y.L.); h.fy157@163.com (F.H.); kangyankai8529@163.com (Y.K.); chenyy2405@163.com (Y.C.); 2 National Medium and Small Leaf Tea Plant Germplasm Resource Repository (Changsha), Changsha 410125, China; 3 National Center for Tea Improvement, Hunan Branch/Hunan Tea Variety and Seedling Engineering Technology Research Center, Changsha 410125, China

**Keywords:** metagenomics, amplicon sequencing, soil microbe, organic fertilizer, soil properties

## Abstract

Fertilization is an essential aspect of tea plantation management that supports a sustainable tea production and drastically influences soil microbial communities. However, few research studies have focused on the differences of microbial communities and the variation in tea quality in response to different fertilization treatments. In this work, the soil fertility, tea quality, and soil microbial communities were investigated in two domestic tea plantations following the application of chemical and organic fertilizers. We determined the content of mineral elements in the soil, including nitrogen, phosphorus, and potassium, and found that the supplementation of chemical fertilizer directly increased the content of mineral elements. However, the application of organic fertilizer significantly improved the accumulation of tea polyphenols and reduced the content of caffeine. Furthermore, amplicon sequencing results showed that the different ways of applying fertilizer have limited effect on the alpha diversity of the microbial community in the soil while the beta diversity was remarkably influenced. This work also suggests that the bacterial community structure and abundance were also relatively constant while the fungal community structure and abundance were dramatically influenced; for example, Chaetomiaceae at the family level, Hypocreaceae at the order level, Trichoderma at the genus level, and Fusarium oxysporum at the species level were predominantly enriched in the tea plantation applying organic fertilizer. Moreover, the bacterial and fungal biomarkers were also analyzed and it was found that Proteobacteria and Gammaproteobacteria (bacteria) and Tremellomycetes (fungi) were potentially characterized as biomarkers in the plantation under organic fertilization. These results provide a valuable basis for the application of organic fertilizer to improve the soil of tea plantations in the future.

## 1. Introduction

Soil provides an ecosystem platform for nutrient cycling, water regulation, and carbon sequestration [1]. Microorganisms serve as one of the important members of soil function to mediate many ecological responses, such as soil aggregation, soil organic carbon (SOC) decomposition, and nutrient transformation [2,3]. Soil microorganisms provide not only organic compounds and soil stability, but also a promising environment as an excellent indicator of soil health [4]. The negative effect of chemical fertilizer application on microbial community variations has been widely reported and investigated [5,6,7]. For example, the application of chemical fertilizers resulted in changes in biomass and the diversity of the bacterial community in rice fields [8]. And the combined utilization of inorganic fertilizer and organic amendments was proved to be an essential practice of soil management during crop production [9].

Organic farming is gradually expanding all over the world. One of its key points is that crops grow in an organic system which largely depends on the activities of soil microorganisms, particularly in terms of mineral nutrition supply [10,11,12]. A classic example is companion planting, which is a cultivation technique in which different species of plants are planted simultaneously on the same field [13]. It has been suggested that the improvement of yields can be effectively enhanced by means of companion planting using White clover (*Trifolium repens* L.) and Orchard grass (*Dactylis glomerata* L.), owing to the change in nitrogen fixation efficiency caused by the remodeling of nearby microorganisms [14,15]. Another strategy is the application of organic fertilizer. Organic fertilizers are defined as naturally transformed fertilizers and generally include all animal manure such as meat processing waste, slurry, manure, and guano and even plant-derived fertilizers such as compost and biosolids [16]. Previous findings indicated that the application of organic fertilizers causes short- and long-term influences on the soil microbial community structure. For example, the supplementation of organic fertilizers had an influence on the microbial growth and diversity of soil microbial community relative to inorganic fertilization, such as increasing the amounts of Gram-negative bacteria [17]. During rice practice, biogas slurry coupled with chemical fertilizer (BCF) improved the rhizosphere microbial diversity and abundance compared with the application of solely chemical fertilizer [18]. Another work suggested that the application of biogas slurry not only improved the contents of available nutrients but also regulated the microbial community structures [19]. Through 16S rDNA amplicon analysis, it was found that the abundance of microbial functional groups related to the metabolism and circulation of carbon (C), nitrogen (N), and phosphorus (P) and antibiotic biosynthesis significantly increased under long-term organic fertilizer treatment [20]. Another finding showed that microbial biomass and oxidoreductase enzyme activities are tightly correlated with the supplementation of organic municipal solid waste [21]. It has been proved that organic fertilizer can improve the physical and chemical properties of the soil, with higher SOC and available N and P contents than chemical fertilizer, and promote microbial growth and enzyme activity [19,22,23]. Furthermore, the bacterial richness and diversity of microorganisms potentially contributed to the production of high-quality crops [24].

Tea is a popular, healthy, caffeine-containing beverage. According to statistics, 3 billion people consume tea daily around the world [25]. Amino acids, polyphenols, and caffeine are the main metabolites that determine the taste and quality of tea. China is the largest producer of tea, planting more than 3.4 × 10^6^ ha and harvesting 14.5 million tons of tea, based on the record of FAO in 2022 (https://www.fao.org/faostat/en/#home, accessed on 26 April 2024). Organic tea plant cultivation provides an alternative method, which reduces the eutrophication risk, and has been widely practiced to improve production quality and soil fertility [26,27,28]. Furthermore, the long-term application of organic fertilizers significantly decreased heavy metals accumulation in rhizosphere soil and tea leaves [29]. Although the use of organic fertilizers could improve the yield of tea leaves and soil properties, there is little information concerning the biomass and diversity changes of microbial community in tea plantations using chemical fertilizers and organic fertilizers. Herein, we first compared the soil nutrients and quality indicators of tea during the long-term use of organic and chemical fertilizers in two tea gardens, and the amplicon sequencing technology was further used to determine the effects of organic and chemical fertilizers on soil microorganisms. The specific aim of this work was to determine how the supplementation of organic fertilizers and chemical fertilizers influenced the soil microbial community structure in two tea orchards.

## 2. Materials and Methods

### 2.1. Study Area

All experiments were performed at the Hexizhen (HXZ) tea plantation and the Qingancun (QGC) tea plantation in Jishou City, Hunan Province, China (28.18° N, 109.43° E). Both tea plantations are located in an area with a subtropical monsoon humid climate, an annual temperature of 17.3 °C, and an annual mean precipitation of 1358.6–1552.5 mm. Both HXZ and QGC are seven-year-old tea plantations, and similar agronomic management practices were performed. Two different fertilization methods were carried out in the HXZ and QGC plantations. In HXZ (organic treatment), a fermentation product (organic fertilizer) of soybean meal, furfural residue, and straw (0.3 kg/m^2^) was applied by ditching in October. Meanwhile, green manure was interplanted every year. In QGC (inorganic treatment), a potassium sulfate compound fertilizer with fertilizer efficiency > 54% (N:P_2_O_5_:K_2_O:MgO = 18:8:12:2; 0.07 kg/m^2^) was applied by ditching in October every year.

### 2.2. Tea Sampling and Analysis

The material used in this work was ‘Baojing Huangjincha 1’ cultivar (*Camellia sinensis* L.). Young leaves from the HYZ and QGC stations were sampled to determine quality. All leaves were frozen in liquid nitrogen and stored at −80 °C until metabolite analyses. The water extract was determined through boiling water reflux [30]. The contents of tea polyphenols, amino acids, and theanine were measured using the HPLC system based on published descriptions [31]. The content of caffeine was also determined by means of the HPLC method according to the caffeine determination method published in a previous study [32].

### 2.3. Soil Sampling and Analysis

All samples were collected randomly at 5 different points. About 400 g of soil sample was collected longitudinally at 0–20 cm and 20–40 cm in depth at the HYZ and QGC stations, respectively. The samples were air-dried naturally and then ground through a 60-mesh sieve after the removal of impurities. For each sample, three replicates were conducted. All samples were stored at −80 °C until use. The pH value was determined using Orion Lab Star PH111 pH Bench Meters (Thermo Scientific, Waltham, MA, USA). The soil organic matter content was measured according to the description published in a previous study [33]. The soil total nitrogen content was determined using the Kjeldahl method [34]. Hydrolysable nitrogen content was determined using the alkali-hydrolyzed diffusion method [35]. The content of available potassium and phosphorus in soil was determined according the methods described by Silva et al. [36].

### 2.4. DNA Extraction and Library Construction

The soil genome DNA was extracted using a DNA Extraction Kit according to the manufacturer’s descriptions. And the purity and integrality were monitored by means of agarose gel electrophoresis and spectrophotometry using a NanoDrop spectrophotometer (Eppendorf, Hamburg, Germany). According to the concentration, DNA was further diluted to 1 ng·µL^−1^ and instantly stored at −20 °C until further use. For amplicon generation, the hypervariable regions 3 to 4 (V3–V4) of 16S rRNA gene were amplified using the specific primer pairs of 338F/806R (338F: 5′-ACTCCTACGGGAGGCAGCA-3′; 806R: 5′-GGACTACHVGGGTWTCTA AT-3′) [37]. For the identification of fungi, internal transcribe spacers (ITSs) were amplified used specific primer pairs of ITS3 (5′-GCATCGATGAAGAACGCAGC-3′) and ITS4 (5′-TCCTCCGCTTATTGATATGC-3′) [38]. All PCR reactions were carried out with 25 µL of Phanta SE Super-Fidelity DNA Polymerase (Vazyme, Nanjing, China); 0.2 µM of forward and reverse primers; and about 50 ng template DNA. The PCR reaction program consisted of pre-denaturation at 98 °C for 3 min, followed by 30 cycles of denaturation at 98 °C for 10 s, annealing at 50 °C for 30 s, and elongation at 72 °C for 30 s. Finally, an elongation at 72 °C for 5 min was followed by storage at 4 °C. Amplicon quality was estimated by means of gel electrophoresis and purified using a SanPrep Spin Column & Collection Kit (Sangon, Shanghai, China). Then, another round of PCR was performed. For library construction and sequencing, amplicon libraries were constructed using TruSeq^®^ DNA PCR-Free Sample Preparation Kit (Illumina, San Diego, CA, USA) following the manufacturer’s descriptions, and index codes were simultaneously added. The library quality was evaluated based on the Qubit@ 2.0 Fluorometer (Thermo Scientific, Waltham, MA, USA) and Agilent Bioanalyzer 2100 system (Agilent, Santa Clara, CA, USA). Finally, the library was sequenced on an Illumina NovaSeq platform, and 250 bp paired-end reads were generated.

### 2.5. Quality Control of Raw Sequencing Data

The raw data were converted into the original rRNA sequence in FASTQ file format [39]. The raw tags were conducted under specific filtering conditions to obtain high-quality clean data for further analyses according to the Trimmomatic software (version 0.39) [40]. The tags were mapped to the reference database (Silva database, https://www.arb-silva.de/, accessed on 5 September 2023) using the UCHIME algorithm to filter and remove chimera sequences [41]. Nearly all the reads with a reasonably high quality were eligible for further analysis.

### 2.6. Operational Taxonomic Units (OTUs) Cluster and Species Annotation

Uparse software (https://drive5.com/uparse/, accessed on 5 October 2023) was used to sort the high-quality sequence of the valid tags, and the sequences with ≥97% similarity were assigned to the same OTUs. The representative sequence of each OUT was filtered for further annotation. The Silva Database (http://www.arb-silva.de/, accessed on 5 September 2023) was used based on the Mothur algorithm to annotate taxonomic information based on each representative sequence [42]. The OTU abundance information was normalized using a sequence number standard corresponding to the samples with the fewest sequences. Subsequent analyses of alpha diversity and beta diversity were all performed basing on these output-normalized data.

### 2.7. Alpha Diversity Analysis

Alpha diversity refers to the mean diversity in species in different sites or habitats within a local scale [43]. In this work, the differences in the estimated abundance of microhabitats were tested using the Kruskal–Wallis significance test [44] for all pairwise combinations. We calculated 2 induces, including Chao1 and Shannon, to present the species diversity in each sample, and displayed them using R software (Version 4.2.0).

### 2.8. Beta Diversity Analysis

Beta diversity is used to perform a comparative analysis about the microbial community diversity of different samples. Firstly, according to the species annotation results and the OTU abundance information of all samples, the OTU information of the same classification is associated and processed to obtain the species abundance information. At the same time, the systemically occurring relation between OTUs is used to further calculate the Unweighted Unifrac distance [45,46]. In this study, principal coordinate analysis (PCoA) was used based on Unweighted Unifrac to uncover the beta diversity among different groups.

## 3. Results

### 3.1. Chemical Characteristics of Soil and Tea Quality

**Figure 1:**
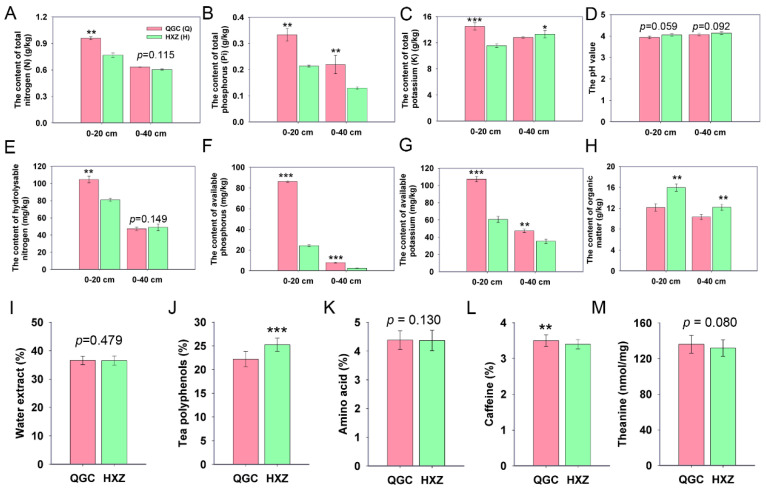
The soil and tea chemical characteristics under organic and chemical fertilizer treatments. (**A**,**E**) The contents of total nitrogen and hydrolysable nitrogen; (**B**,**F**) the contents of total phosphorus and available phosphorus; (**C**,**G**) the contents of total potassium and available potassium; (**D**) the pH value; (**H**) the content of organic matter; and (**I**–**M**) the content of water extract, tea polyphenols, amino acid, caffeine, and theanine of tea. “*”, *p* < 0.05; “**”, *p* < 0.01; “***”, *p* < 0.001.

To effectively evaluate the influences of organic and chemical fertilizers on chemical characteristics in both tea orchards, the soil chemical properties derived from different depth (0–20 cm and 0–40 cm) were determined. Significant differences were observed in the tea orchards treated with either organic (HYZ) or chemical fertilizers (QGC). The results showed that the mineral elements of the soil are mainly enriched in the surface of the soil (0–20 cm) (Figure 1). In general, the contents of total nitrogen, total phosphorus, total potassium, hydrolysable nitrogen, available phosphorus, and available potassium were significantly higher in the chemical fertilizer treatment at a depth of 0–20 cm compared to the organic fertilizer treatment group (Figure 1A–C,E–G). However, the soil pH level was similar between the two tea orchards (*p* > 0.05), both in the surface and deeper in the soil (Figure 1D). As expected, an abundant accumulation of the organic matter was found in the organic treatment group (Figure 1H). However, the contents of total nitrogen and hydrolysable nitrogen were similar between the two treatment groups at a depth of 0–40 cm (*p* > 0.05) (Figure 1A,E).

We also analyzed the chemical characteristics and found that the usage of organic fertilizer significantly increased the content of tea polyphenols and reduced the content of caffeine (Figure 1J,M). However, there is no statistical significance (*p* > 0.05) in the contents of water extract, amino acids, and theanine between the two treated orchards (Figure 1I,K,M).

### 3.2. Sequence Evaluation and Species Annotation

Based on the analyses of soil chemical characteristics, we discovered that the differences of mineral elements in the soil were mainly distributed at the surface soil (0–20 cm) and the subsurface soil (20–40 cm). Therefore, in the present study, we focused on bacterial and fungal sequences in 0–20 cm and 20–40 cm layers between the HXZ and QGC orchards. We defined the samples derived from the HXZ orchard as HA and HB at 0–20 cm and 20–40 cm, and the samples derived from the QGC orchard as QA and QB at 20 cm and 40 cm, respectively. According to the results, a total of 4,160,469 high-quality raw tags were generated, of which 2,148,614 were from full-length 16S rRNA and 2,011,855 were from ITS fragments in both orchards (Tables S1 and S2). After filtering and assembling, on average, 65,212 effective tags from 16S rRNA were obtained and the average length was 254 nt (Table S1). For ITS rRNA sequences, on average, 63,990 effective tags were obtained and the average length was 235 nt (Table S2). The average values of Q20 and Q30 were more than 99% and 97%, respectively (Tables S1 and S2). The effective tags of all samples were arranged from 60.4 to 93.24% in 16S rRNA and ITS sequencing (Tables S1 and S2). Those results showed that the data of this work have good stability and reproducibility.

**Figure 2:**
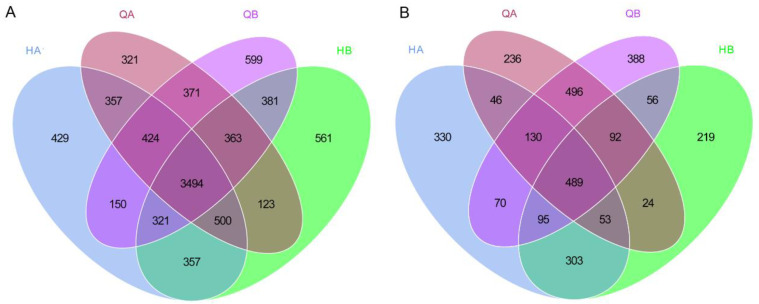
Venn diagram summarizing the unique and overlapping OTUs. (**A**) The total number of OTUs of bacteria was counted and compared among different soil samples. (**B**) The total number of OTUs of fungi was counted and compared among different soil samples.

In order to investigate the soil microorganisms of each sample, effective tags were used to conduct operational taxonomic unit (OTU) clustering based on 97% consistency and species annotation. According to the clustering analyses, there were a total of 8451 bacterial OTUs from full-length 16S rRNA and 3027 fungal OTUs from ITS. Overall, 3494 bacterial OTUs (41.3%) were shared in all samples (Figure 1A) and 429 (5.1%), 321 (3.8%), 599 (7.1%), and 561 (6.6%) bacterial OTUs were detected in HA, QA, QB, and HB samples, respectively (Figure 2A). For fungal taxa, 330 (9.4%), 236 (6.8%), 388 (11.1%), and 219 (6.3%) OTUs were independently detected in HA, QA, QB, and HB samples, of which 489 OTUs (14.0%) were found in all samples (Figure 2B).

### 3.3. Alpha Diversity of the Soil Microbial Community

**Figure 3:**
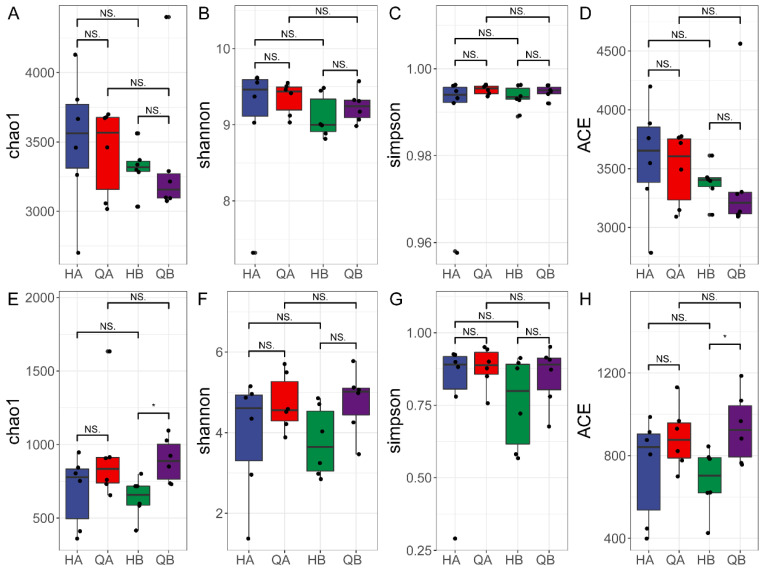
Alpha diversity of microbiomes in organic (HA and HB) and chemical fertilizer (QA1 and QB1) treatment soil. (**A**–**D**) The alpha diversity analysis indices of bacterial species; (**E**–**H**) the alpha diversity analysis indices of fungi species. The alpha diversity indices used in this study were chao1, Shannon, Simpson, and ACE. The datasets from six independent replicates for each sample were pooled. “*” shows statistically significant difference based on a *t*-test (*p* < 0.05); “NS.” means no statistical difference based on a *t*-test (*p* ≥ 0.05).

To determine the complexity of species diversity within the soil samples, the alpha diversity analysis indices of different samples under the consistency threshold of 97%, including chao1, Shannon, Simpson, and abundance-based coverage estimator (ACE), were calculated. No difference in bacterial composition was observed between samples from the two treatment groups (*p* > 0.05) (Figure 3A–D). Those results implied that the organic fertilizer treatment (HA and HB) had no impact on the diversity of soil bacteria. The alpha diversity of fungal composition was analyzed and significantly higher Chao1 and ACE values were only found in the chemical fertilizer treatment group at 0–40 cm (QB and HB, *p* < 0.05) (Figure 3E–H). Overall, the results showed that different ways of applying fertilizer have a limited effect on the alpha diversity of the microbial community in the soil.

### 3.4. Beta Diversity of Microbial Composition

**Figure 4:**
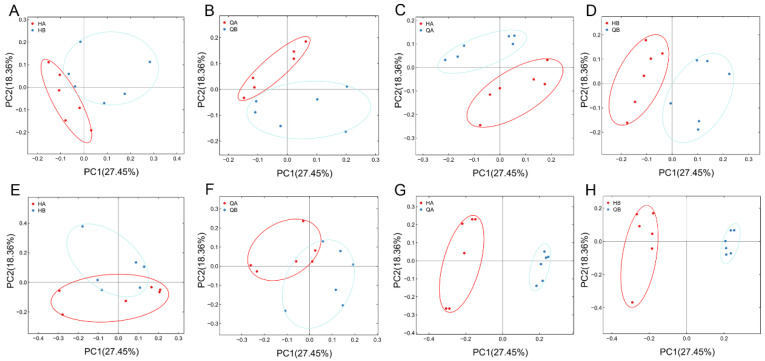
PCoA of bacterial (**A**–**D**) and fungal (**E**–**H**) composition using unweighted UniFrac metrics. In the PCoA of bacteria and fungi, the results of clustering among different samples are shown.

Based on the ANOSIM analysis, the inter-group differences were found to be higher than the intra-group ones (R-value > 0) between 16S rRNA and ITS rRNA, and the statistical analysis was significant (*p*-value < 0.05) except for QB-HB in the bacterial group and QB-QA in the fungal group (Table S3). To investigate the microbial community composition of different samples, principal coordinate analysis (PCoA) was employed to characterize differences in microbial composition between the treatment groups. The results showed that the PC1 and PC2 components of PCoA accounted for 27.45% and 18.36% of the total bacterial and fungal composition variations, respectively. The results showed that the microbial composition of different samples from the same treatment (HA and HB, QA and QB) revealed a slight difference among samples (Figure 4A,B,E,F), while the microbial composition of different samples from the different treatment (HA and QA, HB and QB) was significantly clustered into two separate groups (Figure 4C,D,G,H).

### 3.5. Bacterial Community Structure and Abundance

**Figure 5:**
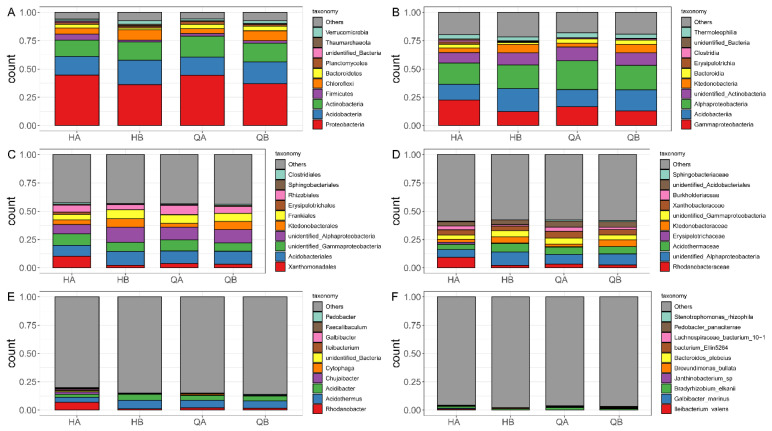
Bacterial composition of the top 10 abundances at different taxonomic levels. The relative abundances (%) of the top 10 most abundant microorganisms at the phylum (**A**), class (**B**), order (**C**), family (**D**), genus (**E**), and species (**F**) level.

To understand the bacterial community structure and abundance, the bacterial community composition at different taxonomic levels was summarized according to the top 10 microorganisms in abundance (Figure 5). The results showed that specific phyla, such as Proteobacteria, Acidobacteria, and Actinobacteria, and specific classes, such as Gammaproteobacteria, Acidobacteriia, and Alphaproteobacteria, had top abundances in all samples and reflected the background bacterial distribution pattern (Figure 5A,B and Table S4). In the HA sample, we found that a unique bacterial distribution at the order, family, genus, and species level, such as Xanthomonadales at the order level, Rhodanobacteraceae at the family level, *Rhodanobacter* at the genus level, and *Ileibacterium valens* at the species level (Figure 5C–F). Compared with the HA sample, the bacterial abundance was different at the class, order, and genus level (Figure 5B,C,E). Similar bacterial structure and abundance are observed between the HB and QB samples (Figure 5).

### 3.6. Fungal Community Structure and Abundance

**Figure 6:**
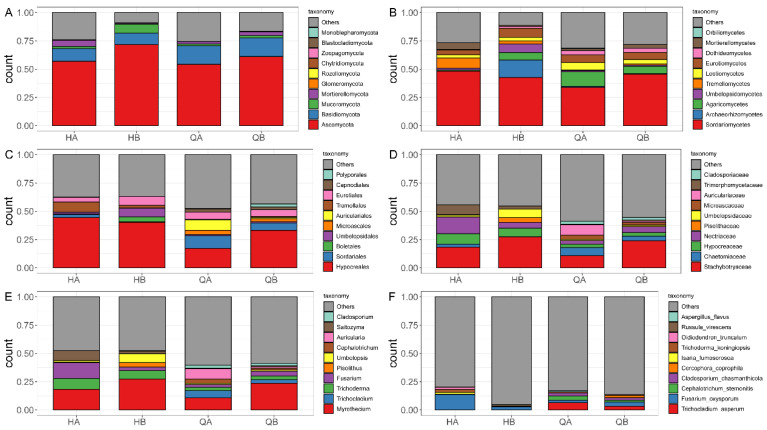
Fungal composition of the top 10 abundances at different taxonomic levels. The relative abundances (%) of the top 10 most abundant microorganisms at the phylum (**A**), class (**B**), order (**C**), family (**D**), genus (**E**), and species (**F**) level.

To further explore the fungal distribution pattern and abundance, the composition of the fungal community at different taxonomic levels according to the microorganisms with the top 10 abundances were summarized (Figure 6). The results indicated that Ascomycota and Basidiomycota served as the basic composition pattern at the phylum level (Figure 6A and Table S5). Compared to the QA and QB samples, a unique class, such as Tremellomycetes, had relatively higher abundance in the HA and HB samples (Figure 6B and Table S5). At the order level, Tremellales was also the dominant taxon in the HA and HB samples. On the contrary, specific orders, such as Microascales and Auriculariales, had significantly higher abundance in the QA and QB samples (Figure 6C and Table S5). We also found the predominant distribution in the HA and HB samples, such as Chaetomiaceae at the family level, *Trichoderma* at the genus level, and *Fusarium oxysporum* at the species level (Figure 5C–F and Table S5).

### 3.7. Effect of Soil Properties on Microbial Communities

**Figure 7:**
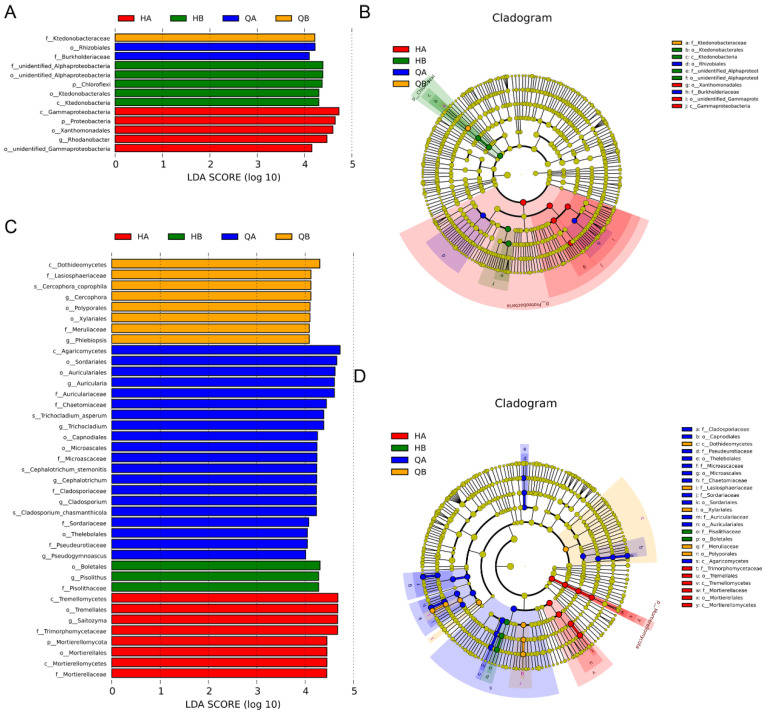
The linear discriminant analysis effect size (LEfSe). (**A**) The LDA values of characteristic bacteria taxa (LDA score > 4). (**B**) The phylogenetic diagram of characteristic bacteria taxa. (**C**) LDA scores of characteristic fungi taxa (LDA score > 4). (**D**) The phylogenetic diagram of characteristic fungi taxa.

To investigate the biomarkers and different levels of taxa change among different soil sample, we performed linear discriminant analysis effect size (LEfSe), which is usually used to uncover high-dimensional biomarkers. According to the results, four bacterial taxa in the HA group with LDA scores greater than 4, namely, Gammaproteobacteria, Proteobacteria, Xanthomonadales, and Rhodanobacter. In the QA group, the orders with LDA scores greater than 4 were Rhizobiales and Burkholderiaceae. The phylum with an LDA score greater than 4 in HB was Chloroflexi. In the QB group, only Ktedonobacteraceae was characterized as the biomarker with an LDA score greater than 4 (Figure 7A,B). A MetaStat analysis was performed to further investigate the bacterial community changes with treatments of organic fertilizer and chemical fertilizer. The results suggested that the abundance of some bacterial taxa at different levels was significantly verified in the soil with different treatment and soil depth. For example, at the phylum level, the Bacteroidetes and Firmicutes in the HA group were significantly up-accumulated compared to other groups (Figure S1). Notably, at the genus level, the change pattern of the bacterial community was significantly verified between HA and QA (Figure S1).

LEfSe analysis was also used to cover different levels of taxa change of fungi among different soil samples and find the biomarkers. The results showed that, in the HA group, Pisolithaceae, Tremellomycetes, Tremellales, Saitozyma, and Trimorphomycetaceae had a higher abundance with LDA scores over 4. In addition, more taxa had LDA scores above 4. In the HB and QB groups, three and eight biomarkers had LDA scores above 4, respectively (Figure 7C,D). At the phylum level, according to the MetaStat analysis, the results indicated that the abundance of Mucoromycota and Ascomycota was increased. Moreover, many fungi with higher relative abundance at the genus level were characterized in the HA group, such as Cephalotrichum, Trichocladium, and Pseudogymnoascus. On the contrary, these specific taxa, which were up-accumulated in the HA group, were significant decreased in the QA and HB groups (Figure S2).

### 3.8. Network Analysis of Microbial Communities

**Figure 8:**
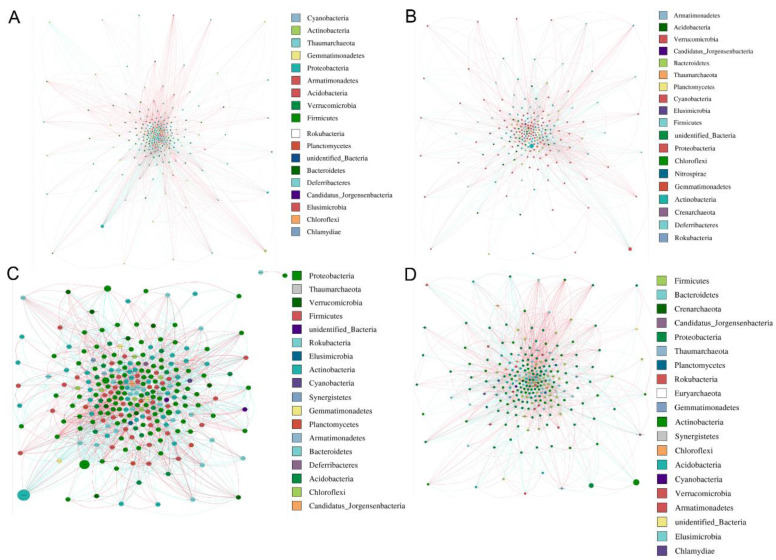
The network of bacterial communities. (**A**–**D**) The network of bacterial communities in the HA, HB, QA, and QB groups. Different nodes refer to different genera; the size of nodes reflects the average abundance of this genus; the color of the lines represents the positive or negative correlation (red is positive correlation; blue is negative correlation).

**Figure 9:**
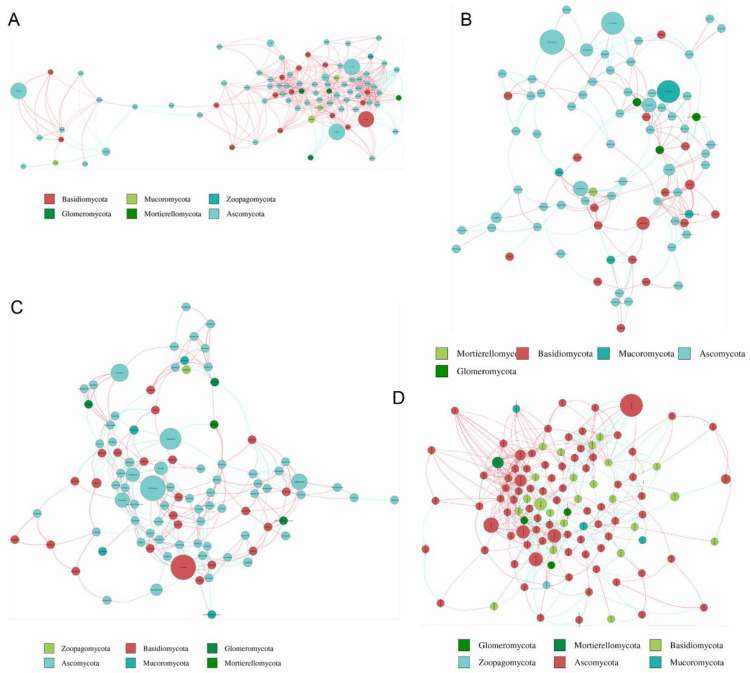
The network of fungal communities. (**A**–**D**) The network of fungal communities in the HA, HB, QA, and QB groups. Different nodes refer to different genera; the size of nodes reflects the average abundance of this genus; the color of the lines represents the positive or negative correlation (red is positive correlation; blue is negative correlation).

Based on calculation of the Spearman correlation coefficient, the microbial networks in four groups from HA, HB, QA, and QB were generated. The results showed that the core phyla of bacteria and fungi were Proteobacteria and Ascomycota, respectively (Figure 8 and Figure 9). In addition, we summarized the topological properties of the bacterial networks and found that the topological characteristics, including modularity (MD), network diameter (ND), clustering coefficient (CC), graph density (GD), average degree (AD), and average path length (APL), in the four groups did not significantly change, except for the fact that AD in HA is higher than that of other groups (Table S6). For fungi, CC, GD, and AD in the HA group had much higher values than those of other groups (Table S6). The results indicated that the network relationship of the fungal communities was probably influenced by the application of the organic fertilizer.

## 4. Discussion

Long-term practice has proved that the utilization of organic fertilizer is an effective way to improve and sustain soil fertility and crop yield [47,48]. Tea is one of the most fashionable nonalcoholic beverages, obtained from the tender leaves of the tea plants (*Camellia sinensis* L.) after processing. Previous findings suggested that the soil fertility of tea orchards and tea quality, including the accumulation of amino acids and phenolic compounds, decreased annually with increasing years of monoculture and the utilization of chemical fertilizers [49]. Our results showed that the contents of nitrogen (N), phosphorus (P), and potassium (K) were significantly higher in the chemical fertilizer treatment (QGC) than in the organic fertilizer treatment (HXZ) (Figure 1). These results indicated that chemical fertilizer directly provides nutrient elements in tea orchards. It was putatively argued that organic fertilizer needs more time to degrade while chemical fertilizer quickly dissolves into the soil [50]. However, another investigation suggested that the long-term supplementation of organic fertilizer effectively provides the N, P, and K contents, much as using chemical fertilizer [29]. This difference may be due to the inconsistent duration of organic fertilizer application. In addition, soil acidification, which is usually caused by the excessive use of nitrogen fertilizers, has become another problem for agriculture [51]. However, our data showed that organic fertilizer application barely improved soil pH (Figure 1D). In fact, the soil pH variation is not aways consistent after the application of organic fertilizer. For example, sewage sludge was used as fertilizer in agriculture and caused the soil pH to decrease [52]. Another two-year field experiment proved that the application of organic fertilizer increased the soil pH value [53]. Therefore, the type of organic fertilizer and the fertilization cycle may be the cause of the pH change. We further evaluated tea quality by determining the characterized compounds and found that the organic fertilizer treatment (HXZ) significantly increased the content of tea polyphenols, while it decreased the content of caffeine, which may interfere with sleep (Figure 1J,L) [54]. Many studies revealed that the long-term application of organic fertilizer significantly improved the growth and quality of tea [55,56]. Our results were consistent with previous findings that the organic fertilizer treatment increased the contents of tea polyphenols and other beneficial metabolites, which was potentially caused by the accumulation of micronutrients [29]. In previous findings, the content of micronutrients was positively correlated with catechin contents [57].

The loss of microbial diversity and balance potentially leads to significantly harmful consequences for ecosystem processes [58]. According to the statistics of the alpha index of each sample, only the Chao 1 and ACE indices of fungal diversity of QB were higher than those of HB (Figure 3). Those results suggested that the application of the organic fertilizer did not change the bacterial diversity in terms of species richness, potentially due to the short application cycle of the organic fertilizer in this study [59]. The PCoA analyses suggested that the application of the organic fertilizer strongly influenced variation in bacterial and fungal community structure. In particular, the HA and HB groups differed from the QA and QB groupd (Figure 4), which may be because the surface of the soil is more susceptible to fertilization. It was suggested that the soil chemical properties potentially contributed to the significant variations in microbial structure among all groups [60].

The samples from different groups have different microbial composition. In terms of bacterial community structure, our results showed that Proteobacteria, such as Gammaproteobacteria and Alphaproteobacteria, Acidobacteria, and Actinobacteria were the most abundant phyla in all samples (Figure 5A,B), which was consistent with previous findings that Proteobacteria and Acidobacteria were predominant bacterial taxa in tea orchard soil systems [61]. Proteobacteria exist widely in soil; for example, 10–30% of soil bacteria belong to Proteobacteria in different producing ecoregions [62]. However, significant differences in soil bacterial structure were scarcely observed at the phylum and class levels among different samples (Figure 5A). In addition, the high abundance of Xanthomonadales at the order level and *Rhodanobacter* (belonging to Rhodanobacteraceae) at the genus level were observed in the HA soil group (Figure 5C–E). However, other studies also found that Xanthomonadales and *Rhodanobacter* significantly decreased in 10-year and 20-year tea orchards or suppressive soil [61,63]. This work further found that fungal diversity was significantly different between organic and chemical fertilizer treatments. The most abundantly identified fungal phyla were Ascomycota and Basidiomycota (Figure 6A). At the class level, Tremellomycetes and Archaeorhizomycetes were typically characterized in the HA and HB groups, respectively. Moreover, Tremellales, Nectriaceae, and *Saitozyma* increased in abundance in the tea orchard with organic fertilizer application. We also identified the fungi order (Hypocreaceae) whose abundance increases with organic fertilizer application (Figure 6C,D). These results suggested that the application of organic fertilizer in tea orchards has a positive role in the improvement of microorganism abundance [64].

Furthermore, we obtained characterized bacterial and fungal biomarkers for each group through LEfSe analysis (Figure 7). In terms of bacterial biomarkers, only two biomarkers (Rhizobiales and Burkholderiaceae) and one biomarker (Ktedonobacteraceae) were identified in the QA and QB groups, respectively. Previous studies suggested that Rhizobiales belong to the *α*-proteobacteria and contain many taxa that form symbiotic relations in plants; they typically serve as a source to perform N_2_-fixation [65]. It was speculated that the abundance of Rhizobiales might be partially contributed to the higher N accumulation in the QGC tea orchard (Figure 1A). In the HA and HB groups, Proteobacteria were obtained as specific biomarkers, which in consistent with the idea that Proteobacteria belong to oligotrophic organisms which usually live in nutrient-poor environments such as deep oceanic sediments, glacial ice, and deep undersurface soil [66]. Moreover, Gammaproteobacteria were also characterized as biomarkers in the HA group, consistently with previous findings that Gammaproteobacteria can utilize a wide array of organic compounds, produce diverse secondary metabolites and antibiotics, and inhibit plant and animal pathogens; it was speculated that the application of organic fertilizer facilitates the occurrence of beneficial taxa. Thanks to the noteworthy differences in fungal community structure, fungal biomarkers were also found among all groups. Consistently with the above results, Tremellomycetes (Tremellales) showed significant enrichment in the HA group. According to previous findings, some species of Tremellomycetes have extreme environmental tolerance and antimicrobial effects, and usually serve as opportunistic pathogens [67]. In the QA group, 16 biomarkers had LDA scores above 4, such as Agaricomycetes, Sordariales, and Auriculariales. Those results suggested that the composition of soil microbial communities has been obviously varied. Although variations in the abundance of these biomarkers were identified in the soil of tea orchards, their ecological function is still elusive.

The stability of microbial communities is commonly affected by changes in the environment. Co-occurrence networks served as a useful method to uncover the interaction and co-existence modes among the myriad microbes, which helps to understand the changes in microbial structure in the soil [68]. Our results showed that the key phylum of bacteria did not change in the two tea orchards with the application of organic or chemical fertilizer. And Proteobacteria account for a larger proportion in all groups (Figure 8). Previous research proved that Proteobacteria can use unstable carbon sources to produce exopolysaccharides to bind sand grains [69]. We also found links, and the average degree was not fluctuating among all groups (Table S6). Therefore, with the application of organic fertilizer, the stability and complexity of bacterial community were not significantly affected. In terms of fungal stability, our results showed that the key phylum of tea orchards was Ascomycota (Figure 9). Based on the results of topologic parameters, the average degree (AD), clustering coefficient (CC), and graph density (GD) were much higher in the HA group than in the other groups (Table S6). Thus, in the soil of the HA group, a more complex fungal community was potentially constructed again during the application of organic fertilizer. It is reported that the application of chemical fertilizer and organic fertilizer had different effects on rhizosphere fungi species. For example, the organic fertilizer was beneficial for the growth of Ascomycota and Olpidiomycota, but the chemical fertilizer significantly increased the abundance of Alternaria and Fusarium, which served as fungal phytopathogens [70]. Therefore, both the diversity and abundance of fungi were affected by fertilization.

## 5. Conclusions

In this study, the chemical characteristics of tea plantation soil to which chemical fertilizer and organic fertilizer were applied were examined. Our results proved that although the contents of N, P, and K were directly increased by the application of chemical fertilizer, the quality of tea was improved by the application of organic fertilizer. Furthermore, our results also showed that that different ways of applying fertilizer have limited effects on microbial diversity in terms of species richness in soil, while the soil fungal structure pattern was significantly influenced. And some beneficial fungi, such as Tremellales, Nectriaceae, and Saitozyma, were predominantly enriched in the tea plantation with organic fertilizer application. This study has reference significance for guiding the soil improvement of degraded tea gardens and further improving the growth and quality of tea.

## Data Availability

The datasets analyzed during the current study are available in the NCBI, and the accession number is PRJNA948706 (https://www.ncbi.nlm.nih.gov/bioproject/PRJNA948706, accessed on 9 April 2024) and PRJNA948709 (https://www.ncbi.nlm.nih.gov/bioproject/PRJNA948709, accessed on 9 April 2024).

## Supplementary Materials

The following supporting information can be downloaded at: https://www.mdpi.com/article/10.3390/genes15050610/s1, Figure S1: MetaStat analysis of bacterial abundance from phylum level to species level in organic (HA and HB) and chemical fertilizer (QA and QB) treatment soil; Figure S2: MetaStat analysis of fungi abundance from phylum level to species level in organic (HA and HB) and chemical fertilizer (QA and QB) treatment soil; Table S1: The quality control of 16S sequencing; Table S2: The quality control of ITS sequencing; Table S3: The ANOSIM analyses of sample groups; Table S4: Bacterial community structure and abundance in soil sample; Table S5: Fungal community structure and abundance in soil sample; Table S6: The key characteristics of network analyses in microbial communities.
