# Supplementary figures and images for: Soil Microbial Community Characteristics and Their Effect on Tea Quality under Different Fertilization Treatments in Two Tea Plantations
Source: Genes (Basel). 2024 May 11;15(5):610. doi: 10.3390/genes15050610 (PMC11121415; doi:10.3390/genes15050610)

## Phylum

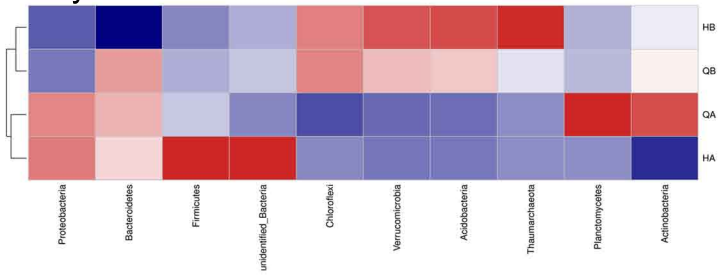

## Class

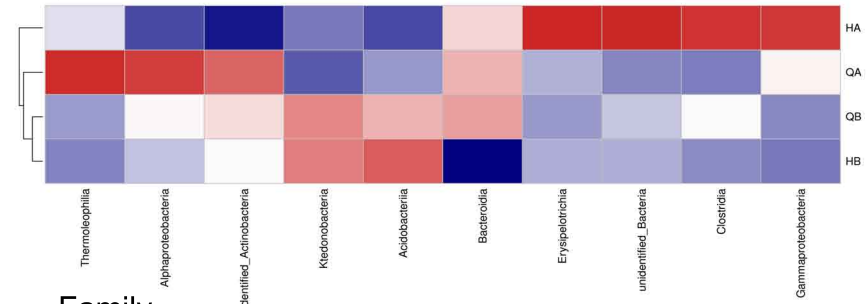

## Order

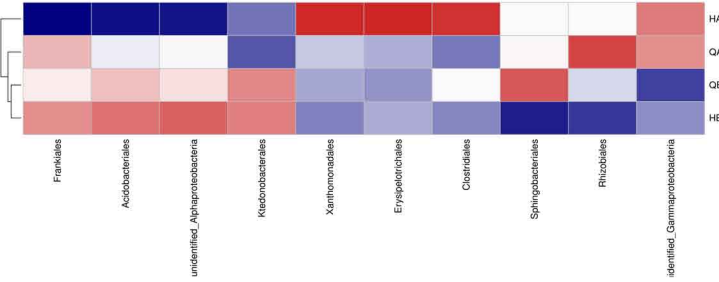

## Family

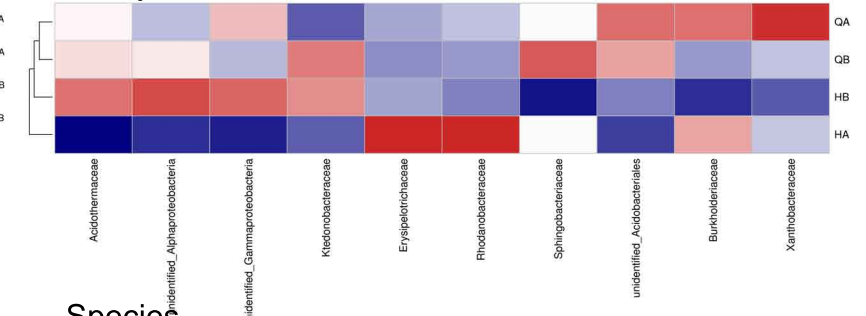

## Genus

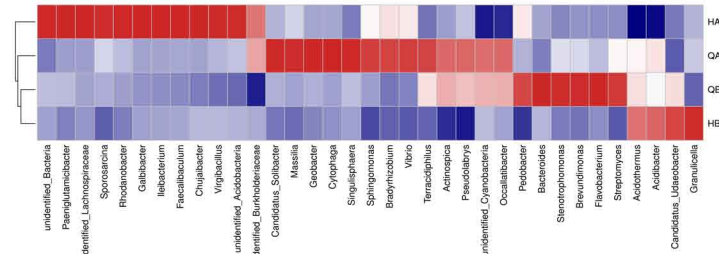

## Species

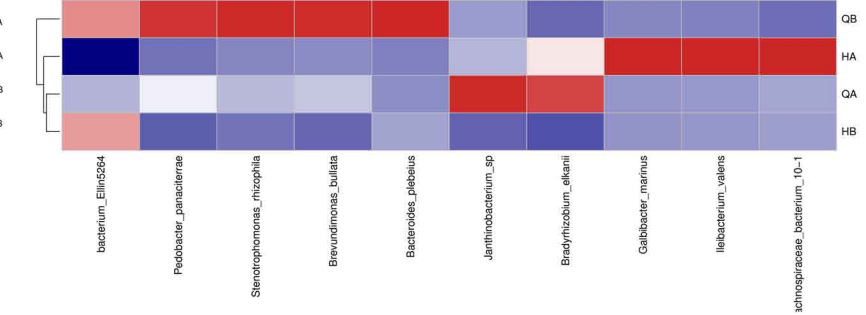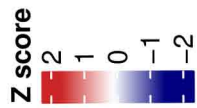

Supplement: Supplementary file 1 [file genes-15-00610-s001.zip › Figure S1.pdf]

## Phylum

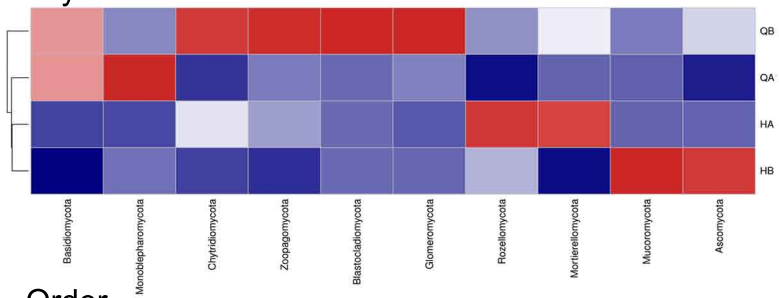

## Class

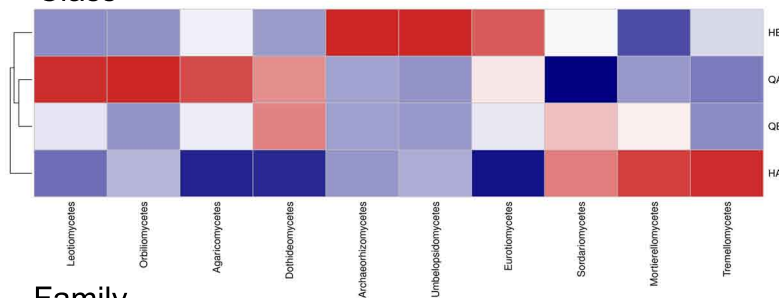

## Order

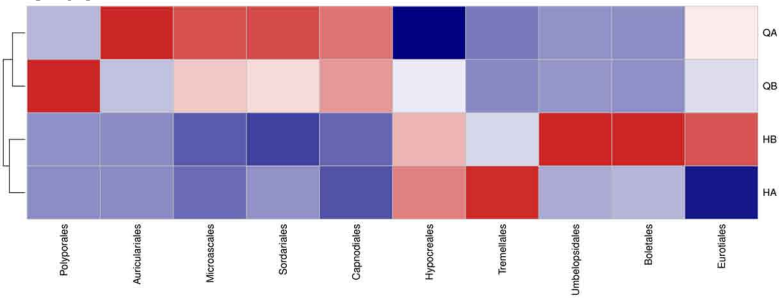

## Family

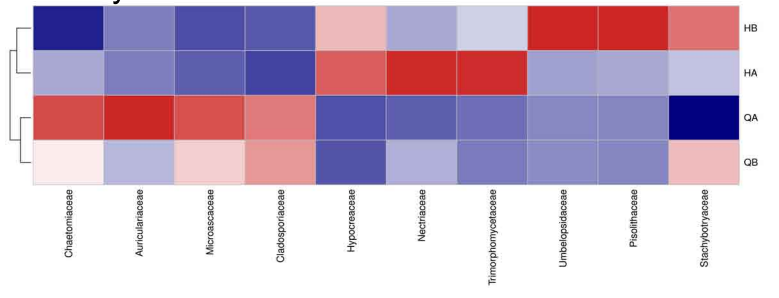

## Genus

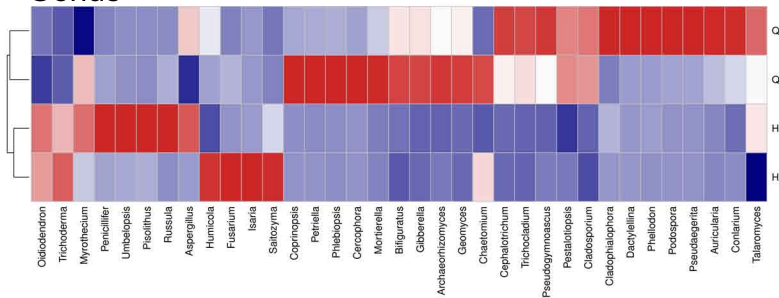

## Species

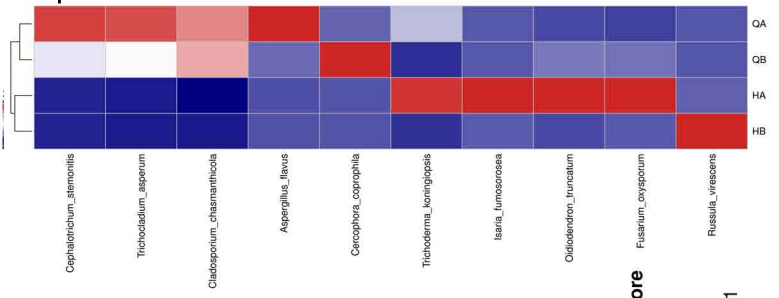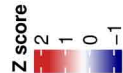

Supplement: Supplementary file 1 [file genes-15-00610-s001.zip › Figure S2.pdf]
